# A randomised study on the comparative performance of German paramedics after technical versus non-technical skills training

**DOI:** 10.3205/zma001858

**Published:** 2026-06-15

**Authors:** Hendrik Eismann, Jörn Halser, Mathias Robert, Markus Flentje

**Affiliations:** 1 Hannover Medical School, Department of Anaesthesiology and Intensive Care Medicine, Hannover, Germany; 2 German Red Cross, Rescue Service, Uelzen, Deutschland

**Keywords:** training, medical service personnel, non-technical skills, medical simulation

## Abstract

**Background:**

The working environment of pre-hospital emergency medicine represents the framework conditions of high-risk organisations. Non-technical skills are an important foundation for successful performance. In Germany, a new job profile - advanced paramedic - was introduced in 2014, which for the first time also included non-technical skills in the curriculum. Another special feature is that for the first time it is regulated how paramedics proceed with selected extended procedures, such as the application of selected medication. The advanced paramedic has a mandatory annual training requirement of 30 hours. Since there are no requirements in terms of content, we investigated whether training of non-technical or technical skills improve the care of a simulated patient. The results should indicate which priorities should be set in training.

**Methods:**

Teams were randomised while training and debriefing took place depending on the study arm with a focus on technical procedures and non-technical skills. Six months later, we evaluated the performance of the teams in a simulation scenario using “time-key-item-product” score and the use of incident management methods.

**Results:**

Comparing performance with crisis resource management (CRM)/ non-technical skills and technical training, we found no significant difference in time-key-item-product score (TKIP). There was also no difference in the application of incident management methods. Checklists were only used in small numbers. Over half of the teams failed to complete 75% of the required procedures in the scenario.

**Conclusion:**

It is noticeable that only a few groups carried out all required procedures. The incomplete treatment suggests that the use of checklists can help emergency teams. Dealing with non-technical skills should be trained intensively, as their use does not yet appear to be routine.

## Zusammenfassung

**Hintergrund:**

Das Arbeitsumfeld der prähospitalen Notfallmedizin stellt die Rahmenbedingungen einer Hochrisikoorganisation dar. Nicht-technische Kompetenzen sind eine wichtige Grundlage für erfolgreiche Teamleistungen. In Deutschland wurde 2014 ein neues Berufsbild – Notfallsanitäter:in – eingeführt, für das erstmals auch nicht-technische Kompetenzen in den Lehrplan aufgenommen wurden. Erstmals wurde geregelt, wie Rettungsfachpersonal bei ausgewählten erweiterten Maßnahmen, wie beispielsweise der Verabreichung ausgewählter Medikamente, vorgehen. Notfallsanitäter:innen müssen jährlich 30 Stunden Fortbildung absolvieren. Da es keine inhaltlichen Vorgaben gibt, haben wir untersucht, ob die Ausbildung nicht-technischer oder technischer Kompetenzen die Versorgung eines simulierten Patienten verbessert. Die Ergebnisse sollen Aufschluss darüber geben, welche Schwerpunkte in der Fortbildung gesetzt werden sollten.

**Methode:**

Die Teams wurden während der Fortbildung randomisiert und die Nachbesprechungen fanden je nach Studienarm mit Schwerpunkt auf nicht-technischen Fähigkeiten oder Algorithmen basierten technischen Fertigkeiten statt. Sechs Monate später bewerteten wir die Leistung der Teams in einem Simulationsszenario anhand des „Time-Key-Item-Product“ Scores und der Anwendung von Methoden des Incident Managements.

**Ergebnisse:**

Beim Vergleich der Leistung mit Crisis Resource Management (CRM)/ nicht-technischen Fähigkeiten und technischer Ausbildung fanden wir keinen signifikanten Unterschied im Time-Key-Item-Product score (TKIP). Auch bei der Anwendung von Methoden des Incident Managements gab es keine Unterschiede. Checklisten wurden nur in geringer Anzahl verwendet. Über die Hälfte der Teams schaffte es nicht, 75 % der geforderten Maßnahmen im Szenario durchzuführen.

**Schlussfolgerung:**

Es fällt auf, dass nur wenige Gruppen alle geforderten Maßnahmen durchgeführt haben. Die unvollständige Behandlung lässt darauf schließen, dass die Verwendung von Checklisten Notfallteams helfen kann. Der Umgang mit nicht-technischen Fähigkeiten sollte intensiv trainiert werden, da deren Einsatz noch nicht routinemäßig zu erfolgen scheint.

## Background

Emergency pre-hospital care is a prime example of a special field of work. Extensive stress of an emergency, time pressure, and limited information are some factors that negatively affect the quality of patient care. From a psychological perspective, this environment is described as a High-Risk Organization (HRO) [1]. Non-technical skills (NTS) were proven to improve the outcomes in an HRO setting [2]. Non-technical and technical skills belong to different categories. NTS includes skills such as task management, situational awareness, teamwork, and decision making. In contrast, technical skills describe the practitioner’s ability to perform procedures such as intubation, thoracotomy, etc. [3], [4]. “To err is human” was the first of multiple publications that discussed avoidable errors and their prevention [5], [6], [7]. Some of these publications highlighted the lack of NTS and considered its improvement as a factor that could be developed even in experienced emergency teams [8], [9], [10]. Training programs that focus on NTS are called crisis resource management courses (CRM), analogous to the Crew Resource Management courses used in the context of aviation [11]. Although CRM training is already integrated into various emergency service curricula, it remains unclear whether the competences developed during the course remain a part of everyday practice or require ongoing enhancement. Simulated scenarios can determine whether such ongoing NTS training is necessary [12], [13]. A scalable indicator of medical care quality is the ratio of completed medical interventions to the recommended actions outlined in standard operating procedures. Studies have shown that up to 23% of the steps are not taken during emergencies [14]. 

European emergency medical services (EMS) contain a variety of job titles – the German and French systems are emergency-physician-based, whereas the Paramedic-focused systems are prevalent in English-speaking countries [15]. The German training program for non-physician emergency employees was reformed in 2014 [https://www.gesetze-im-internet.de/notsang/BJNR134810013.html]. A new job profile of a three-year trained Advanced Paramedic (NotfallsanitäterIn) was introduced, replacing the two-year trained Emergency Medical Technician (EMT) paramedic (RettungsassistentIn). During the study, practitioners of both advanced job profiles took the leading role, assisted by EMT basic practitioners (RettungssanitäterIn). The different tasks of the professional groups are shown in table 1 (Tab. 1).

German districts are required to appoint a physician as the medical director (Ärztliche Leitung Rettungsdienst) of the emergency medical service, who is responsible for the training and education of non-physician emergency employees. According to the Standard Operating Procedures (SOP), the medical director defines the type and scope of the extended procedures that the trainees are required to master. The medical director is also in charge of a regular quality assessment of the emergency-service staff. In previous studies, we’ve found that the professional recommendations, training, and testing significantly differ across various districts of Germany [16], [17]. The current heterogeneity of didactic approaches highlights that a standard approach is yet to be established. The CRM is already incorporated into curricula for emergency service personnel. However, it is unclear whether one-time training is enough to integrate the skill set into everyday work or if repeated NTS training is necessary. Therefore, continuous evaluation and scientific monitoring are crucial in developing the best path to practice.

This study aimed to compare the efficacy of a CRM/NTS training and algorithm-based technical skill training by assessing patient care in a simulated environment. Considering the current literature, we hypothesized that the CRM group would provide faster and more complete patient care due to the application of non-technical skills. The study findings will guide decision-makers in prioritizing the type of training in future curricula to provide optimal patient care.

## Materials and methods

### Study design

Our study was a randomized intervention study. All study participants received the same training on respiratory distress and were then able to apply the learned material in simulated scenarios. They were then assigned to one of two study arms. The first arm received a simulated scenario-based training with a technical debriefing, while the participants in the second arm received a theoretical review about CRM with a debriefing focusing on non-technical skills. On the first day, the training scenarios were all focused on respiratory distress, as further outlined in table 2 (Tab. 2). The clinical scenarios that were later incorporated into the test scenarios were practiced on day one. All test scenarios on day two were identical for all groups.

**Figure 1:**
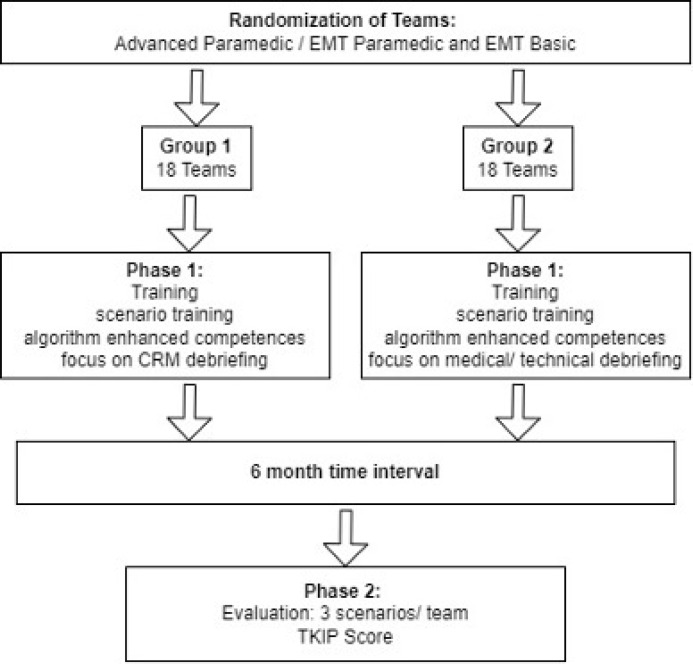
Flow chart of the study. The debriefing only includes the non-technical skills in one study arm EMT: emergency medical technician, CRM: crisis resource management, TKIP: Time-key-item-product score

Six months after the initial training, all teams were evaluated by three pre-programmed simulated scenarios (SimDesigner, Laerdal Medical, Norway) (see figure 1 (Fig. 1)). The scenario outlines and controlling instructions are shown in the Appendix. No further training or simulated scenarios took place between the initial training and the evaluation phase.

### Setting and population

We enrolled 36 active EMS teams from the operational area of Uelzen, Germany. The study of Haerkens et al. was used as the basis for the number of cases (power 80%) [9]. The rescue service had a total of 125 employees. All participants gave written informed consent, and participation in the study was voluntary. The training (see table 2 (Tab. 2)) was included in the mandatory annual EMS training in Germany.

The teams consisted of an advanced EMT practitioner (either an EMT paramedic or an advanced paramedic) as a team leader and a EMT basic participant. All activities adhered to the standard operating procedures and were chosen by the EMS medical director. The participants were randomly assigned to two groups: the technical debriefing and the non-technical debriefing study arms, using an online randomization tool [https://www.randomizer.org/]. Advanced paramedics and Basic-EMT worked together in their original team constellations. The time frame in our study adhered to the time frames used in other studies [10] – all teams received a one-day (eight hours minus breaks) simulation-based training in the German Red Cross training facility in Uelzen, Germany. In Germany, a one-day course is required for continuous medical education. Many providers choose this format, and it has been utilized in several studies. All scenarios were carried out using a SimMan Essential patient simulator (Laerdal Medical, Norway), a fully equipped rescue backpack (Pax Wasserkuppe, X-CEN-TEK, Germany), a Medumat Standard ventilator and oxygen module (Weinmann Emergency Medical Technology GmbH, Germany), and an Accuvac Rescue suction device (Weinmann Emergency Medical Technology GmbH, Germany) usually utilized in the EMS of the participants. All participants received an introduction to the features of the patient simulator.

The teams were debriefed according to the study arms, either with a focus on technical skills and crisis resource management (CRM) or medical topics and therapy algorithms (TD). The technical debriefing focused on the adherence to standard operational procedures and covered the medical background of every scenario. The non-technical debriefing was composed of a set of impulse questions for each scenario and a debriefing based on the CRM guiding principles by Gaba et al. [18].

### Data collection

The teams were evaluated in three scenarios, six months after the initial training, with a total of 108 scenarios performed. This six-month timeframe was chosen, as it allows evaluation of long-term retention and content application [19], [20]. Advanced paramedics and EMT paramedics were appointed as team leaders, having the same professional competencies and real-life duties throughout the study period. Each scenario was recorded as multiscreen synchronized videos using DSone (ZYOS GmbH, Germany). Demographic data of the teams were gathered via questionnaires, while performance data were evaluated from the recorded videos. The data were analysed by two experienced instructors, who were independent of the training team.

### Rating of team performance

**Figure 2:**
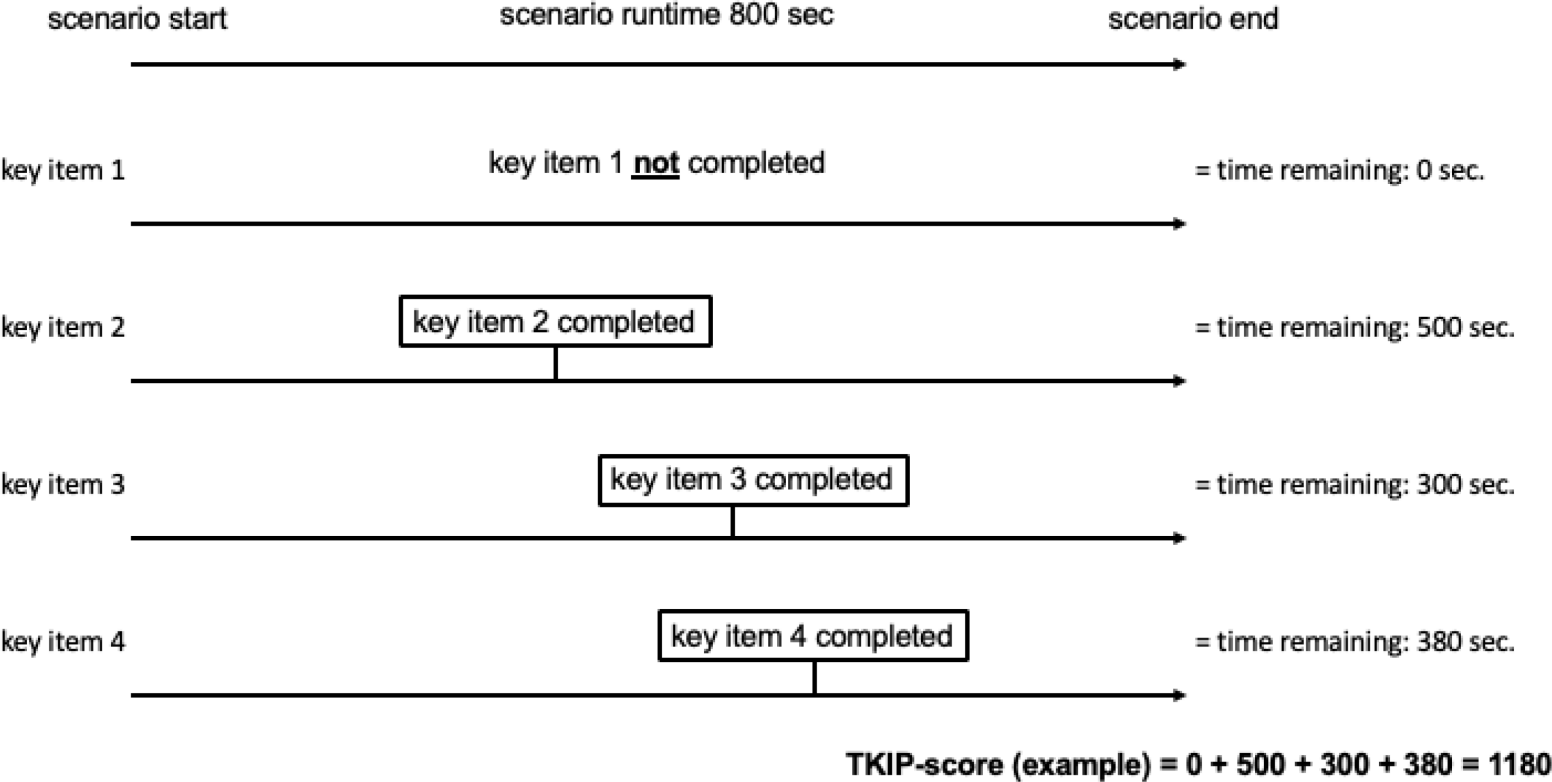
Principle of the TKIP-score (Time-Key-Item-Product score). After the completed care step (key item), the remaining time is calculated to 600 sec. All sum of the remaining times evaluates the quality of care.

The team performance was evaluated by the time-key-item-product score (TKIP), which was developed and used in other studies [21]. The score reflects a comprehensive evaluation of the quality of performance of the necessary procedures and the speed of execution. All necessary care steps were defined for each scenario. In our study, the total scenario duration was set at 600 seconds. If a measure was carried out completely and correctly before the set time, the time of completion was recorded. The remaining time after completion of the procedure, up to 600 seconds, is included in the score. If a measure was not carried out, the value for this item is 0 seconds. The sum of the remaining times from all procedures is used to generate the TKIP score, and the level of the value reflects the quality of care (high TKIP indicates good performance) (see figure 2 (Fig. 2)). The suspected diagnosis is considered to be completed when the diagnosis has been spoken out loud or communicated to the team members. TKIP measurement allows a combined assessment of speed and completeness of patient care. In line with other studies, the reference points of 50% and 75% of the required measures were also presented [21]. To compare different scenarios with a different number of items, the final results were compared using a conversion factor. As an example, the final result of a scenario with eight items will be multiplied by a factor of 1,25 to be compared with a scenario with 10 items. The key items in the scenarios were taken from the treatment guidelines (SOP) of the medical director.

### Rating factors of non-technical skills

As the study results are based on the completeness of the medical procedures, selected factors of non-technical skills were evaluated separately. The following factors were determined by the authors: using/ reviewing checklists, implementing checklists, communicating checklist content, and team communication (10-for-10). The 10-for-10 concept describes a short timeout (10 seconds) in the work process during which all information, ideas, and any concerns are exchanged. Then, the treatment plan for the next 10 minutes is communicated [11]. All videos were analysed by experienced trainers (>10 years of simulation experience, training in accordance with regional German protocol). The Mangold interactive video analysis software was used (Mangold INTERACT Version 2023, Arnstorf, Germany). The number of interactions of non-technical skills was recorded for each scenario and presented as a total.

### Statistics

All collected data were processed with SPSS 26 (IBM, Corporation, USA). Event times of the TKIP-Score were measured using a stopwatch and displayed in Kaplan-Meier curves. Differences were analysed using the log-rank test. All data were tested for normal distribution using the Kolmogorov-Smirnov test. Descriptive data were presented according to frequency. Differences between the study arms were analysed using the Mann-Whitney U test. The Mann-Whitney-U test was also used to analyse differences between the investigated groups according to the frequency of CRM procedures.

## Results

### Demographic data

Of the 36 teams initially trained (18 per group), three were unavailable due to illness and withdrew from the study or job rotation. In phase two, 15 teams per group participated in the evaluated scenarios. The following professionals were assigned: nine advanced paramedics, six EMT paramedics, 15 EMT basic (group “CRM”; seven females, 23 males), and nine advanced paramedics, six EMT paramedics, 15 basic EMT (group “tech”; nine females, 21 males). The mean age of the team leader was 39,2±8,1 years (group “CRM” 47,9±7,5 years; group “tech” 36,5±7,8 years).

### Effect of debriefing on time and adherence to key items and non-technical skills

Comparing performance with CRM and technical training, we found no significant difference in TKIP-Score (see table 3 (Tab. 3)). With this model, all patients were treated with the same quality of care. There was also no difference in the number of non-technical skill methods used in the two study arms (see table 4 (Tab. 4)).

### TKIP score for overall team performance

**Figure 3:**
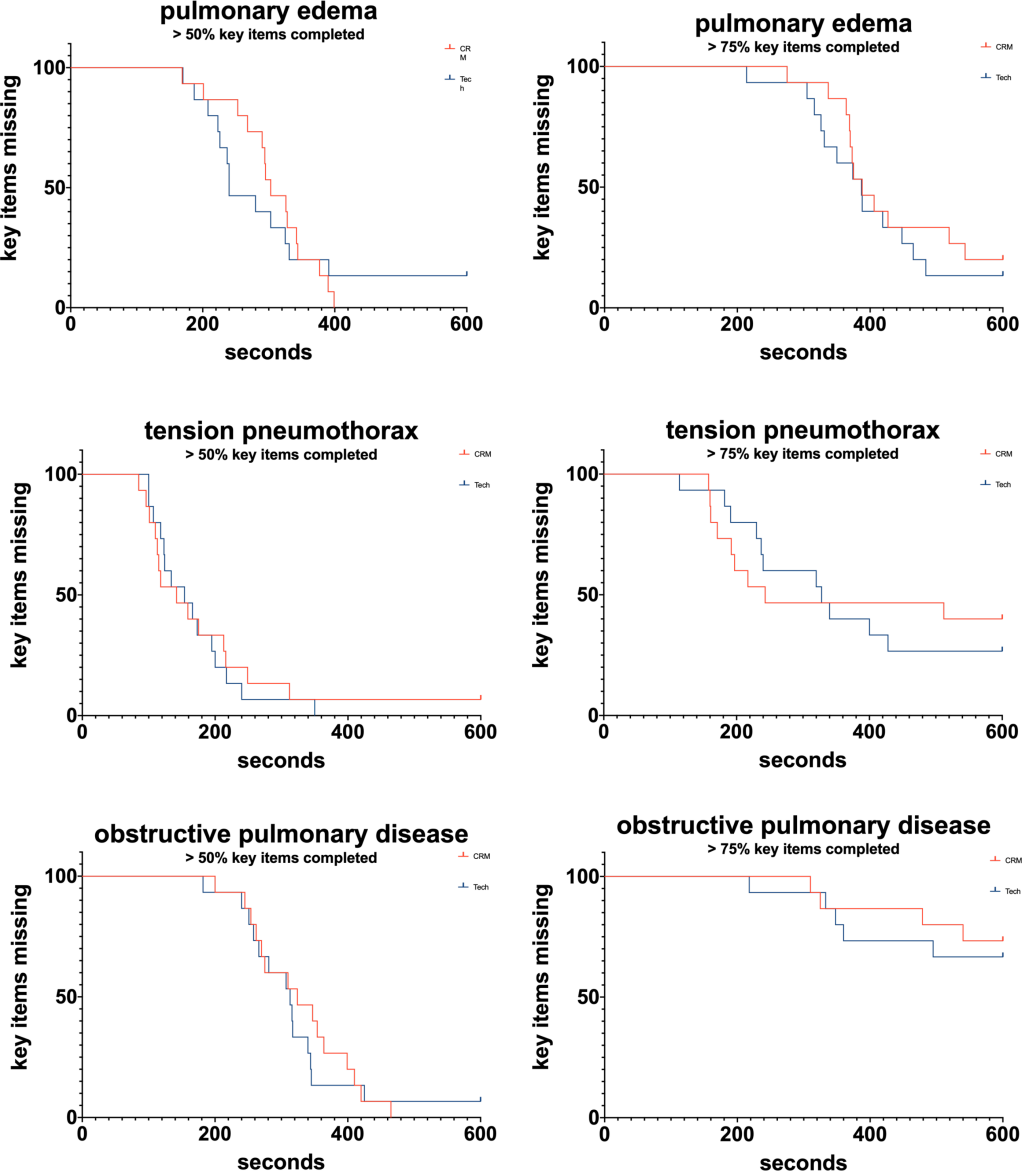
Kaplan Meyer graphs of the simulated scenarios. Key items missing for the groups “>50%” and “>75%” are depicted Type of debriefing is shown in red (crm-debriefing) and blue (tech-debriefing). Log rank test: pulmonary edema 50% p=0,9158; pulmonary edema 75% p=0,4747; tension pneumothorax 50% p=0,7748; tension pneumothorax 75% p=0,7807; obstructive pulmonary disease 50% p=0,6794; obstructive pulmonary disease 75% p=0,6753.

The evaluation of the number of teams with the time used to fulfil 50 or 75% of the required items is shown as Kaplan-Meier graphs in figure 3 (Fig. 3). Not all teams have successfully attained the 75% item completion benchmark. It was apparent that less than half of the teams were able to perform 75% of the required procedures in the “obstructive pulmonary disease” scenario. Overlooked procedures and the frequency of their omission are detailed in table 3 (Tab. 3).

### Effect of CRM use on time and adherence to key items according to type of scenario

In both groups, the defined methods for supporting non-technical skills were described in their number of applications. The Mann-Whitney U test showed no significant difference between the groups for any measure, with the overall number of applications being very low. Even the most applied communication tool (10-for-10) [11] was scarcely used, with approximately one to three teams using this tool. The detailed data is presented in table 4 (Tab. 4).

## Discussion

This study investigated a didactic learning intervention and its effect on patient care in a simulated environment. This study aimed to assess the efficacy of a CRM/non-technical skill training in comparison to algorithm-based technical training and to provide valuable insight to the medical directors for planning and optimizing future regular training programs and curricula. We chose to focus on respiratory distress scenarios, as they are routinely practiced by rescue teams. Relying on an analysis that showed that almost 8.2% of pre-hospital care cases were associated with this presentation [22], we assumed that the study participants had experience in providing pre-hospital care in the said setting. A caveat was thoracentesis, as this procedure was rarely performed [16].

There was no difference between the quality of performance of CRM and tech-group in the delivery of medical procedures. After analyzing the frequency of use of Crisis Resource Management tools, this result does not come as a surprise, as the results of both groups were similarly low. A growing awareness of the importance of CRM and NTS started around the year 2000, and in 2014, they were integrated into the advanced paramedic curriculum. Another development was the possibility for advanced training for EMT paramedics, at the end of which they would become certified advanced paramedics. The training was not regulated, and the trainees’ NTS were evaluated via an oral exam [https://www.gesetze-im-internet.de/notsang/__2a.html]. According to Jünger et al., the oral examination is not a suitable assessment method of application competence [23]. Similar framework conditions also apply to the emergency physician course, in which the CRM content was integrated in 2018 [24]. As in the EMT course, the examination is also conducted as an oral exam. Transferring knowledge from advanced training to everyday life poses a challenge that courses can’t fully meet. A productive approach to integrate the learned material in teams requires both positive support and negative comments [25], meaning that negative feedback should be given when interventions are not applied. In an earlier study about learned skills integration into real-life emergency caesarian sections, we found that feedback on non-applied new content is not frequently given [26]. Our study led us to conclude that the current curricular integration and transfer climate are insufficient for CRM and NTS integration into routine practice in the field – a one-time 8-hour intervention is inadequately short for a change in these framework conditions. Our findings and data interpretation correspond to the current literature, which found that changes in the medical environment take 17 years before being fully integrated [27]. The duration of successfully described trainings in the context of CRM varies from one day to a prolonged concept [10], [28]. It is therefore necessary to analyze the specific target group. Backgrounds are described as multifactorial.

The analysis of the non-implemented procedures in the 75% analysis shows that procedures from both the basic and the extended are not implemented. Other studies have also shown that experienced emergency medicine practitioners do not execute all required measures in an emergency, emphasizing the importance of checklist implementation by all emergency care practitioners [21], [29], [30]. Our study shows that further medical training doesn’t improve CRM skills and that a focused CRM-method-focused training is essential. The data from the scenarios can be used for self-reflection by the teams.

The study participants were aware that they were constantly observed and evaluated, which could result in bias due to the observation effect. Notably, the participants scarcely used any checklists. A point that led us to conclude that if the observation effect was indeed influencing their behaviour, they were convinced that checklist usage was discouraged. Possible reasons for deficient checklist implementation could be cultural acceptance, poor training, and faulty implementation strategies [30]. Additionally, the lack of acceptance for the use of checklists has been established for decades in German emergency medical services training as well as in professional practice. Only relatively recently has “having to look something up” stopped being seen as an admission of weakness. An intervention such as the one-time training in this study design is therefore unlikely to lead to a significant change in the use of checklists.

One of the main takeaways of the studies is the necessity of checklist integration into pre-hospital patient care. According to other studies, this implementation must also include team training, tracking of adoption, resources, and organization [31][. Future studies could evaluate checklist utilization and other incident training methods.

### Limitations

In our study, we applied the TKIP score developed by Just et al. for the first time [21]. Retrospectively, the prioritization of important procedures could’ve been beneficial and could be used as a factor of importance. For example, an important procedure could get the score of two minor procedures. Another important factor that arose during our study is the application of measures that harm the patient. We have not observed such cases in our scenarios, but the application of contraindicated measures should be considered in further studies.

In our study, the teams were not differentiated according to their expertise level in order to simulate the reality of the work environment in an ambulance. It is possible that this team appointment influenced the results. Team structuring could be modified and made according to experience could be beneficial in a target group-oriented learning setting.

The skills that were measured in this study are a part of the professional standard of advanced and basic EMTs, and therefore, a single targeted training that took place six months before the evaluation does not reveal a significant difference. Further studies with more frequent training and review intervals may be more appropriate. Due to the complex study design, the research question could only be fully addressed in a small subset of cases. As a monocentric study, it cannot be assumed that the results represent every rescue service in Germany. As mentioned above, there are major differences in terms of training and organization [17]. Nevertheless, we believe that the results of our study can be used to prioritize training evaluation to describe the situation. The current study in the local area can be used as an example for other medical directors.

## Conclusion

The aim of the study was to provide recommendations for the responsible directors in the emergency services to optimize the regular training programs. The main goal of the study was to assess the efficacy of a CRM/ non-technical skill training in comparison to algorithm-based technical skill training. Our hypothesis that a one-day CRM training course would have a positive impact on patient care has not been confirmed. As in other studies in the field, neither team performed all medical procedures. Despite the acknowledged value of checklists, they remain underused. We conclude that to integrate non-technical skills in a real-life pre-hospital setting, it must be included in the emergency medicine service curricula alongside regular technical medical training.

## Acknowledgements

The authors thank all EMS professionals who participated in this study. We thank Florian Hempel for his support in the study planning. Finally, we thank Michael von Geyso, as Heinz and Tim Meierhoff from the German Red Cross Uelzen, for their support in conducting this study. We thank Alan Gutman for linguistic review. 

## Notes

### Author contributions

All authors listed have contributed sufficiently to the project. HE: conception and design of the study, acquisition of data, analysis and interpretation of data, drafting the manuscript; JH: acquisition of data, drafting the manuscript; MR: acquisition and interpretation of data, drafting the article; MF: conception and design of the study, drafting the manuscript.

### Authors’ ORCIDs

- Hendrik Eismann: [0000-0003-0962-8091]
- Markus Flentje: [0000-0003-3686-8998]

### Ethic

The study was approved by the ethics committee of the Hannover Medical School (no. 3635-2017. 

### Funding

The study was financed with departmental funds.

## Competing interests

The authors declare that they have no competing interests. 

## Figures and Tables

**Table 1:**
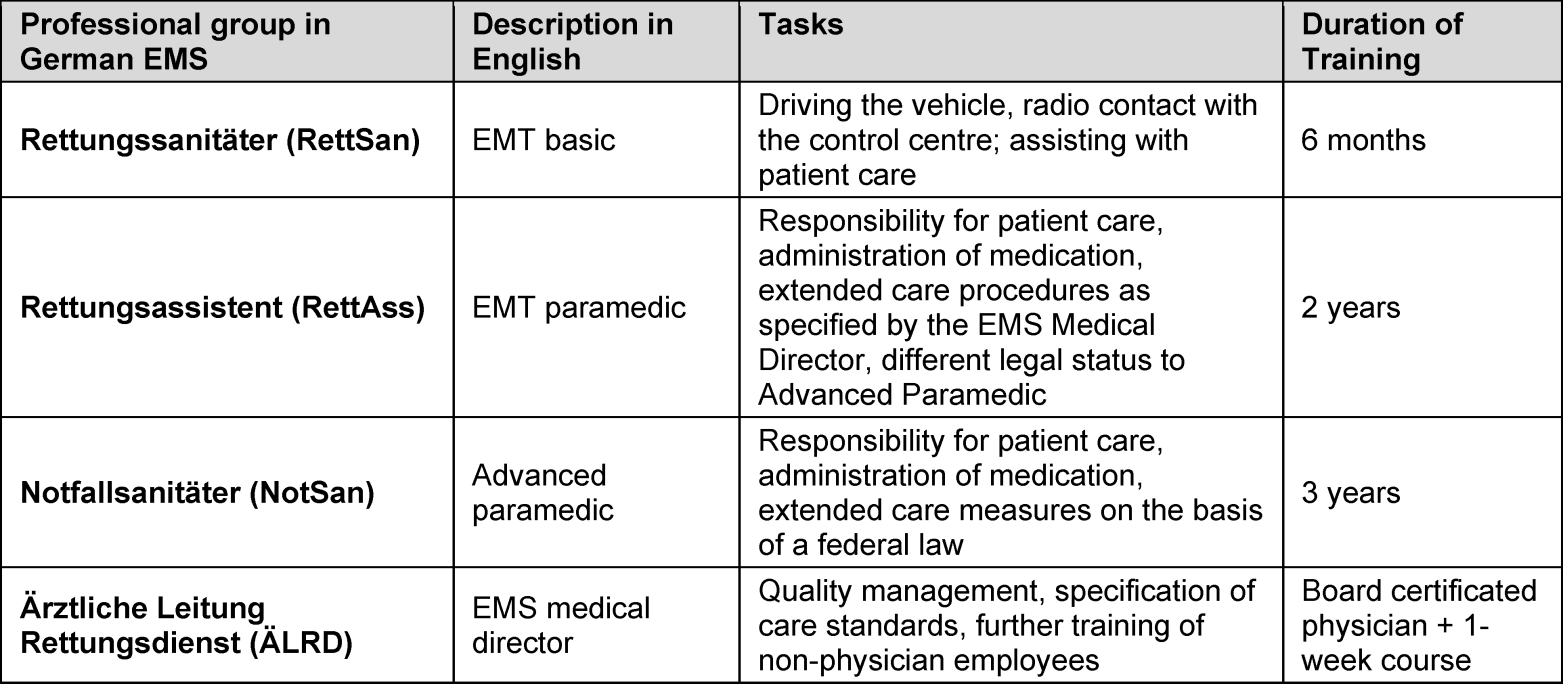
Professional groups and areas of responsibility in the German emergency medical system (without prehospital emergency physicians (Notarzt) EMT: emergency medical technician; EMS: emergency medical system

**Table 2:**
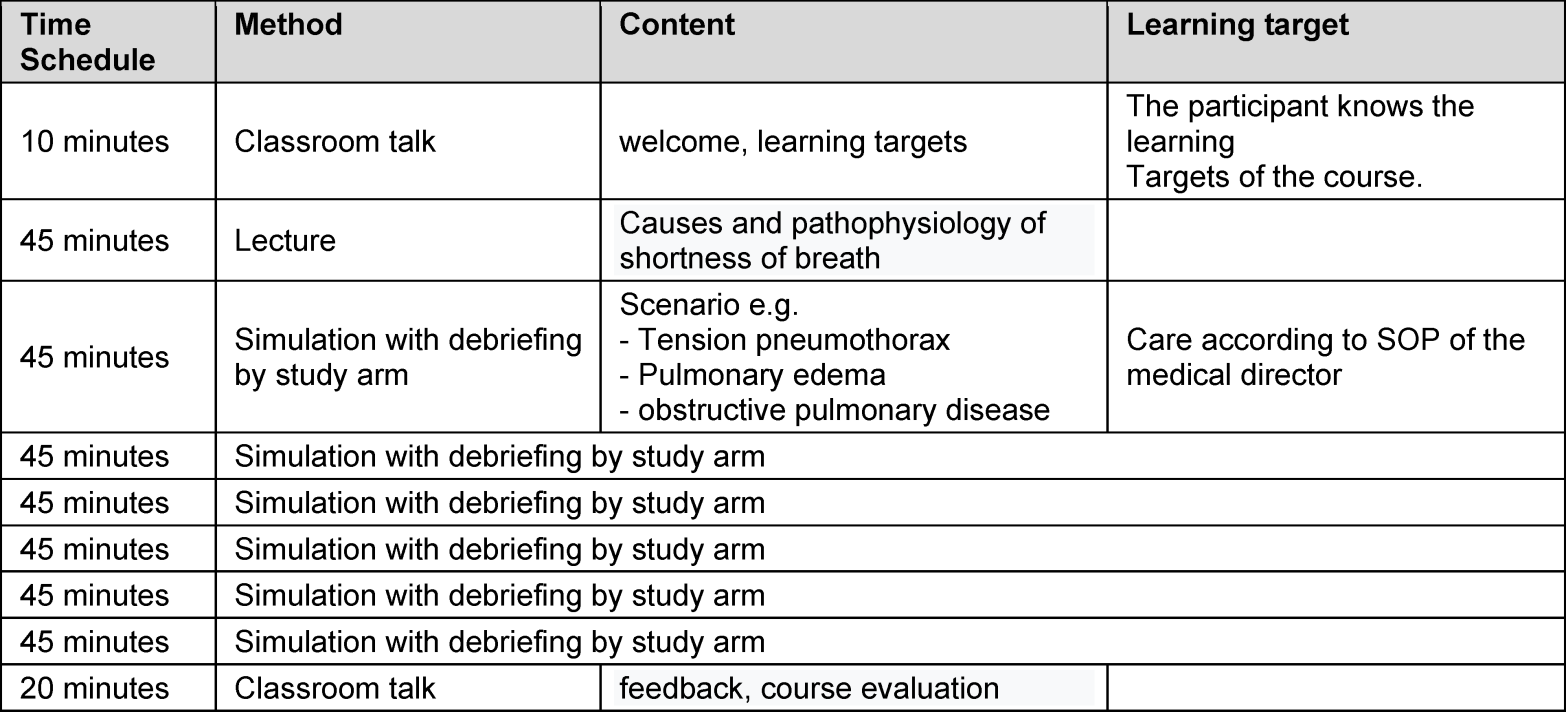
Course agenda for the simulation based-training (day 1 of the study)

**Table 3:**
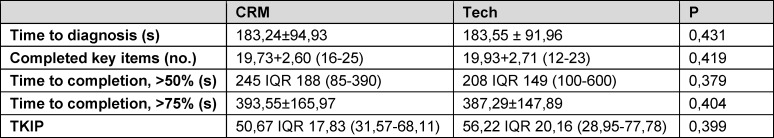
Data are given in mean and SD or median and IQR, minimum and maximum in brackets according to distribution. Kolmogorov-Smirnov test: time to diagnosis p<0,001; time to completion >50% p=0,20; time to completion >75% p<0,001; TKIP p=0,20 CRM: Crisis Resource Management debriefing, Tech: technical debriefing, TKIP: Time-key-item-product

**Table 4:**
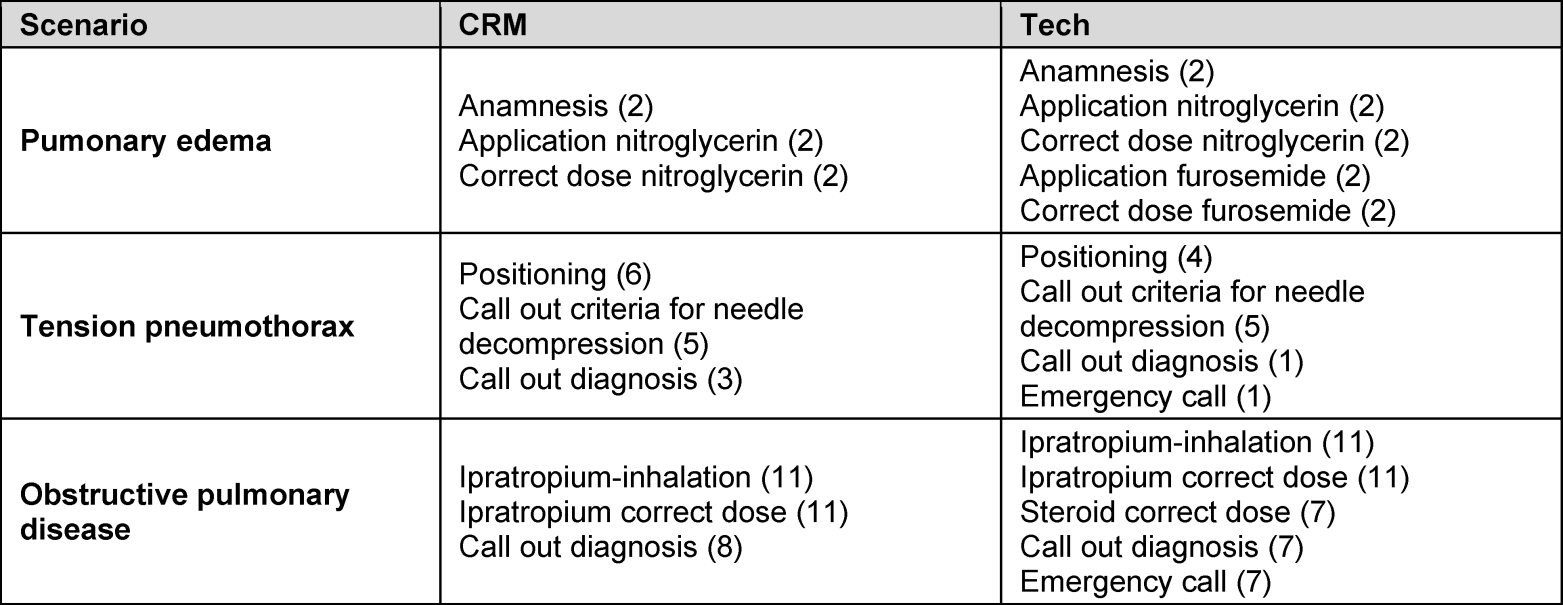
Missing items in the 75% evaluation with frequencies in brackets. Both basic and extended procedures are represented CRM: Crisis Resource Management debriefing, Tech: technical debriefing
